# Lipid Extract from Hard-Shelled Mussel (*Mytilus coruscus*) Improves Clinical Conditions of Patients with Rheumatoid Arthritis: A Randomized Controlled Trial

**DOI:** 10.3390/nu7010625

**Published:** 2015-01-16

**Authors:** Yuanqing Fu, Guipu Li, Xinhua Zhang, Gengyan Xing, Xiaojie Hu, Lifeng Yang, Duo Li

**Affiliations:** 1 Department of Food Science and Nutrition, Zhejiang University, Hangzhou 310058, China; E-Mails: fuyuanqing@163.com (Y.F.); guipuli@aliyun.com (G.L.); 2 Department of Rheumatology, Zhejiang Sir Run Run Shaw Hospital, Hangzhou 310058, China; E-Mail: singwar@163.com; 3 Department of Orthopaedic Surgery, The General Hospital of Chinese People’s Army Police Force, Beijing 100039, China; E-Mail: xgy7766@263.net; 4 College of Life Science, Linyi University, Linyi 276005, China; E-Mail: xjhuhz@126.com; 5 Ningbo College of Health Sciences, Ningbo 315100, China; E-Mail: lifeng_yang@126.com

**Keywords:** anti-inflammation, clinical conditions, hard-shelled mussel, lipid extract, rheumatoid arthritis

## Abstract

Studies have suggested a lipid extract from hard-shelled mussel (*Mytilus coruscus*) (HMLE) possessed strong anti-inflammatory activity in arthritis model of rats. This study investigated whether HMLE could improve clinical conditions of rheumatoid arthritis patients. Fifty rheumatoid arthritis patients (28–75 years) were randomly assigned to receive HMLE capsules or receive placebo capsules for 6 months. Forty-two subjects and 50 subjects were included in per-protocol and intention-to-treat analysis, respectively. Significant differences in changes on disease activity score (DAS28) and clinical disease activity index (CDAI) after 6-month intervention (*p* < 0.01) were observed in both analyses with more evident efficacy shown in per-protocol population (∆DAS28 = 0.47; ∆CDAI = 4.17), which favored the benefits of the HMLE group. TNF-α (tumor necrosis factor α), interleukin (IL)-1β and PGE_2_ (prostaglandin E2) but not IL-6, were significantly decreased in both groups, and the decrements were much larger in the HMLE group for TNF-α and PGE_2_ after 6 months from baseline (*p* < 0.05). IL-10 was significantly increased in both groups and the change was much more evident in the HMLE group (*p* < 0.05). In conclusion, HMLE exhibited benefits for the clinical conditions of rheumatoid patients in relation to improvement in the balance between pro- and anti-inflammatory factors, which indicated its potential to serve as adjunctive treatment for rheumatoid arthritis. (ClinicalTrials.gov NCT02173587).

## 1. Introduction

Rheumatoid arthritis (RA) is a chronic, progressive, systemic autoimmune disorder, characterized by abnormal immune responses, inflammation of joints with severe pain and swelling, and irreversible destruction of cartilage and bone in the joints [1,2,3,4,5]. RA affects between 0.5% and 1% of adults in the developed world, with 5–50 per 100,000 new cases every year [6]. The conventional therapeutic options employed in the management of RA are corticosteroids, immunosuppressive drugs and non-steroidal anti-inflammatory drugs (NSAIDs), but these options frequently produce suboptimal benefits and may be associated with various side effects [7]. It is well known that inflammatory factors regulated a broad range of inflammatory processes that are implicated in the pathogenesis of rheumatoid arthritis. Increased levels of tumor necrosis factor (TNF)-α, interleukin (IL)-1β and IL-6 were found in the joint tissues of patients suffering from RA [8], which indicated the positive association between these inflammatory mediators and RA pathogenesis. TNF-α and IL-1β were reported to be participating in the macrophage-dependent inflammation, and the activation of macrophages led to cyclooxygenase 2 (COX-2) stimulation with consequent prostaglandin E2 (PGE_2_) overproduction [9,10]. TNF-α, IL-1β, IL-6, PGE_2_ and COX-2 played fundamental roles in articular destruction, cartilage degradation, bone erosion and comorbidities associated with RA [8,11]. On the other hand, there are also anti-inflammatory cytokines such as IL-4 and IL-10, which inhibit a range of inflammatory mediators and cytokines including PGE_2_, TNF-α, IL-1β, IL-6, IL-8 and IFN-γ, at both protein and mRNA levels [12,13]. Therefore, the imbalance between pro- and anti-inflammatory cytokines played an important part in the induction of autoimmunity and chronic inflammation in arthritis. Nowadays, targeted biological therapies including anti-TNF inhibitors, IL-1 receptor and IL-6 receptor antagonists, have performed greatly and revolutionizing the treatment of RA. However, the significant cost and infection thereafter are bottlenecks of their extensive application. Therefore, developing functional food or dietary supplements with effective anti-inflammatory activity may be a good addition to current therapeutic options.

Previous work in our laboratory demonstrated that a lipid extract (HMLE) from hard-shelled mussel (*Mytilus coruscus*)—which is the main mussel species widely cultivated in coastal areas of China and the most representative mollusk in the Chinese bivalve market—exhibited strong anti-inflammatory and anti-arthritic activity [14]. In both adjuvant-induced and collagen-induced rat models of arthritis, HMLE supplementation significantly improved paw swelling, arthritic index and body-weight gain. Further examination showed HMLE down-regulated pro-inflammatory factors including PGE_2_, IL-1β, IL-6 and TNF-α, and up-regulated anti-inflammatory cytokines such as IL-4 and IL-10, which indicated the strong efficacy may be attributed to the regulation of balance between pro- inflammatory and anti-inflammatory factors [14]. However, no clinical human study has been conducted to examine the anti-arthritic activity of the HMLE. In the present study, we conducted a randomized placebo-controlled trial to investigate whether HMLE supplementation could improve clinical conditions in patients with active rheumatoid arthritis. Validated disease activity score (DAS28) and clinical disease activity index (CDAI) and their core components such as number of tender and swollen joints, patient’s and physician’s global assessment of disease activity and erythrocyte sedimentation rate (ESR) were used to reflect the clinical disease activity.

## 2. Experimental Section

### 2.1. HMLE Capsule and Corn Oil Capsule Preparation

Hard-shelled mussels of commercial size were collected in November, 2011, from Shengsi Islands in Zhejiang province. The preparation of vacuum freeze-dried powder of hard-shelled mussels and procedures of CO_2_ supercritical extraction were described previously [14]. The resulting lipid extract presented as amber oil and the lipid composition was analyzed using Iatroscan TLC-FID Analyzer (Iatron Laboratories Inc., Tokyo, Japan) as previously reported [15]. Quantification of each lipid class was expressed as relative percentage of total HMLE. The fatty acid compositions of total HMLE and its main lipid classes and placebo were measured by gas chromatography (Shimadzu GC-14C, Shimadzu Corporation, Japan) as described previously [15]. HMLE was stored under −80 °C in amber vials to minimize autoxidation and was mixed with corn oil (Yihai Kerry Co., Ltd., Shanghai, China) before being processed into capsules (Zhejiang Dalishen Health Product Co., Ltd., Hangzhou, China). Each capsule contains 200 mg HMLE and 200 mg corn oil and the safety of capsules was qualified by National Light Industry Food Quality Supervision and Testing Center (Hangzhou, China).

### 2.2. Subjects

Eligible patients had a diagnosis of active RA ≥ 6 months prior to screening based on the criteria of American College of Rheumatology, and the active disease was defined as at least four swollen joints and six tender joints and at least two of the following: ESR > 28 mm/h; morning stiffness lasting ≥45 min and serum C reactive protein (CRP) concentration > 2.0 mg/dL. Major exclusion criteria included ongoing rheumatic or inflammatory joint diseases other than RA; subjects suffering hyperlipidemia, hyperglycemia, diabetes mellitus, cardiovascular diseases, gastrointestinal problems or liver and renal failure; history of malignancy; serious allergies to biological agents or treatment with any investigational agent at less than 4 weeks of screening. All subjects gave their informed consent for inclusion before they participated in the study. The study was conducted in accordance with the Declaration of Helsinki, and the protocol was approved by the Ethics Committee of Biosystems Engineering, Zhejiang University, Hangzhou, China (ZJU-BEFS-2014007).

### 2.3. Study Design

In this randomized placebo controlled study (ClinicalTrials.gov NCT02173587), patients were permitted to maintain their prescribed medications (mainly Methotrexate, Corticosteroids and NSAIDs) and were randomly assigned 1:1 to two groups according to a computer-generated randomization list. Patients allocated in the first group administered orally four HMLE capsules (200 mg HMLE and 200 mg corn oil per capsule) once daily after meals for first 2 months and 2 HMLE capsules once daily after meals for subsequent 4 months. Patients in the second group administered orally placebo capsules (400 mg corn oil per capsule, identical in appearance to HMLE capsules) according to the same schedule. Capsules were dispensed three-monthly in an opaque plastic bottle (coded with A or B) containing 60 capsules and drug compliance was assessed by capsule count and inquiry at each visit. Patients were considered withdrawn if they suspended taking capsules for more than one month (about more than 15% of the expected number of capsules bad not been taken). The allocation groups of the patients were saved by a third party (Ningbo College of Health Sciences) and all subjects, investigators, and outcome assessors will not know the allocation groups of the patients based on the appearance of the given capsules. All patients were asked to keep a 7-day food diary before intervention and during week 12. Dietary energy and nutrient intake of each subject were assessed from the dietary records by using “Diet Analysis” software (Cao Aihong, Taiyuan, China). All the enrolled patients visited Zhejiang Sir Run Run Shaw Hospital in the morning following an overnight fast at baseline and 3-month intervals, and underwent anthropometric measurements including height, body weight, heart rate and blood pressure. Then, clinical evaluations were performed during face-to-face interview by a professional attending doctor and 10 mL of peripheral venous blood was drawn. A full blood examination was performed during the 3 h after blood sampling. Serum and erythrocyte samples were also promptly prepared, portioned into tubes, and frozen at −80 °C until analysis.

### 2.4. Laboratory Measurements and Clinical Evaluation

Fasting blood specimens were obtained from participants at entry, 3 months and 6 months later. CRP, rheumatoid factor (RF) and liver and kidney function parameters were analyzed on HITACHI chemistry analyzer using enzyme-based colorimetric test or colorimetric test supplied by Diasys Diagnostic Systems Co., Ltd. (Shanghai, China) within four hours of blood collection. ESR was analyzed on Monitor 20 (VITAL Diagnostics, Forli, Italy). Levels of inflammatory mediators (PGE_2_) and cytokines (IL-1β, IL-6, IL-10, TNF-α) from serum were determined by research workers who were blind to the patient groups using ELISA Kits (Nanjing Jiancheng Technology Co., Ltd., Nanjing, China) according to the manufacturer’s instructions. Total lipid content of the erythrocyte was extracted with solvents, the methyl esters of fatty acids in erythrocyte were prepared by saponification and the erythrocyte compositions of fatty acids were determined by gas chromatography as described previously [16]. Fatty acids compositions were expressed as a percentage of total erythrocyte fatty acids.

Clinical assessment of each patient was carried out by the same examiner blinded to the allocation sequence at baseline, months 3 and months 6. Validated disease activity score based on 28 joint counts and ESR (DAS28) was a primary outcome measure and the DAS28 was calculated by using the formula: $\text{DASDAS}28=[0.56\times \sqrt{\left(\text{TJC}28\right)}+0.28\times \sqrt{\text{SJC}28}+0.70\times \text{ln}\left(\text{ESR}\right)]\times 1.08+0.16$

(TJC28, Tender Joint Count based on 28-joint assessment; SJC, Swollen Joint Count based on 28-joint assessment; ESR, Erythrocyte Sedimentation Rate) The primary efficacy endpoint was the decline in DAS28 (∆DAS28), which was classified as good (∆DAS28 > 1.2), moderate (0.6 < ∆DAS28 ≤ 1.2), or no response (∆DAS28 ≤ 0.6) according to the European League Against Rheumatism response criteria [17]. The secondary outcome measure was the validated clinical disease activity index (CDAI), which had been proved to be comparable with ACR response criteria [18]. The following formula was used to calculate CDAI: TJC + SJC + PGA_(0–10 cm)_ + MDGA_(0–10 cm)_ + CRP_(mg/dL)._ (PGA, Patients global assessment; MDGA, Physician global assessment; CRP, C-reactive protein) The severity of disease activity was considered as high if the CDAI > 22, moderate if 10 < CDAI ≤ 22, and low if CDAI ≤ 10. Additional endpoints included: tender and swollen joint count based on a 28-joint count, patients’ and physician’s global assessment of disease activity (100 mm visual analog scale, VAS), values of ESR and CRP levels, which were DAS28 or CDAI core component scores; duration of morning stiffness and rheumatoid factor (RF). Safety assessments included incidence and type of adverse events, incidence of intervention-related changes from baseline in liver and renal function parameters, body weight and vital signs. Serious adverse event was classified as fatal or life-threatening, permanently or significantly disabling, requiring hospitalization, involving cancer or suggestive of a significant hazard.

### 2.5. Statistical Analysis

Sample size was based on previous clinical experience in human intervention studies evaluating the efficacy of fish oil on RA [19,20]. The present study was designed to detect a difference in DAS28 decreasing between HMLE and control group of 0.5 (80% of SD). A projected 21 patients were needed in each group to detect the difference with 80% power at two-sided significance level of 0.1. The target number of patients was 50 (25 patients per group) to allow for a dropout rate of 15%. Primary and secondary efficacy analyses were performed on both intention-to-treat (ITT) and per-protocol (PP) populations. For those patients having neither 3 month nor 6 month data, the baseline data was used as the post data in ITT analysis, which means no improvement. For those patients only lacking 6 month data, the 3 month data was used as the post data in the ITT analysis. All other efficacy analyses (mainly DAS28 or CDAI core component scores) were performed on the PP population only. Two-tailed paired *t*-test was used to compare the changes within the same group. Baseline comparisons between groups were compared using chi-square or *t*-test analyses, as appropriate. Repeated measure ANOVA was used to evaluate effects of capsule type, time and the interaction of capsule type and time on tested values. Independent *t*-test was used for comparing the changes of tested parameters from baseline between the two groups. The values of tested parameters were reported as mean ± SEM and changes of tested values from baseline were indicated as mean differences with their 95% confidence intervals (CIs) in tables. Differences were considered significant if *p* < 0.05. SPSS version 16.0 (SPSS Inc., Chicago, IL, USA) was used for data analyses.

## 3. Results

### 3.1. Lipid Profiles and Fatty Acids Composition of HMLE

The lipid profiles of HMLE in present study was similar to lipid extract of Mussels collected during 2005–2006, phospholipids (41.1% ± 1.9%) were the predominant lipid class in HMLE, followed by triacylglycerols (32.5% ± 2.1%) and sterol esters (21.7% ± 2.3%). Small portions of sterols (3.1% ± 0.4%) and free fatty acids (1.6% ± 0.3%) were also observed. Fatty acids composition of HMLE and its components (triacylglycerols, and free fatty acids) and placebo (corn oil) were summarized in Table 1. The fatty acid profiles of total HMLE and its components were basically consistent, with palmitic acid (C16:0, 24.83%–31.37%), docosahexenoic acid (C22:6*n*-3, 18.15%–31.14%) and eicosapentaenoic acid (C20:5*n*-3, 10.37%–13.06%) being the predominant compositions. While the fatty acid composition of placebo was much simpler, and linoleic acid (C18:2*n*-6), oleic acid (C18:1*n*-9) and palmitic acid (C16:0) account for 53.42%, 29.89% and 12.14% of total fatty acids, respectively.

**Table 1:** Fatty acids composition (% of total fatty acids) of HMLE and placebo.

Fatty Acid	Separated from HMLE		Total HMLE	Placebo
	TAG	FFA		
C14:0	3.92 ± 0.04	4.25 ± 0.04	2.71 ± 0.09	ND
C15:0	0.85 ± 0.03	1.02 ± 0.01	0.81 ± 0.06	ND
C16:0	29.00 ± 0.59	31.37 ± 0.46	24.83 ± 0.87	12.14 ± 0.48
C17:0	1.58 ± 0.08	1.55 ± 0.03	2.24 ± 0.09	ND
C18:0	4.45 ± 0.14	3.97 ± 0.06	6.31 ± 0.13	1.70 ± 0.11
Total SFA	39.80 ± 1.01	42.16 ± 0.98	36.90 ± 0.81	13.84 ± 0.41
C16:1	4.08 ± 0.06	6.52 ± 0.06	7.28 ± 0.11	ND
C17:1	0.23 ± 0.01	0.28 ± 0.05	3.10 ± 0.07	ND
C18:1*n*-9	1.08 ± 0.08	1.07 ± 0.04	1.65 ± 0.08	29.89 ± 0.72
C18:1*n*-7	2.00 ± 0.09	1.60 ± 0.07	2.88 ± 0.13	ND
C20:1	1.87 ± 0.11	1.76 ± 0.07	5.81 ± 0.17	ND
Total MUFA	9.26 ± 0.52	11.23 ± 0.44	20.72 ± 0.67	29.89 ± 0.72
C18:3*n*-3	1.66 ± 0.03	1.78 ± 0.04	1.48 ± 0.06	0.70 ± 0.05
C20:5*n*-3	10.37 ± 0.34	11.87 ± 0.29	13.06 ± 0.64	ND
C22:5*n*-3	0.93 ± 0.06	0.82 ± 0.06	0.60 ± 0.05	ND
C22:6*n*-3	31.14 ± 0.67	24.91 ± 0.28	18.15 ± 0.97	ND
Total n-3 PUFA	44.10 ± 1.22	39.38 ± 1.11	33.29 ± 0.96	0.70 ± 0.05
C18:2*n*-6	3.86 ± 0.29	3.98 ± 0.06	2.21 ± 0.09	53.42 ± 1.33
C20:2*n*-6	0.73 ± 0.02	0.74 ± 0.01	0.52 ± 0.05	ND
C20:3*n*-6	0.12 ± 0.01	0.23 ± 0.00	0.45 ± 0.04	ND
C20:4*n*-6	1.12 ± 0.06	1.28 ± 0.02	2.83 ± 0.12	ND
Total *n*-6 PUFA	5.83 ± 0.21	6.23 ± 0.16	6.01 ± 0.21	53.42 ± 1.33

Data are presented as mean ± SEM. TAG, triacylglycerol; FFA, free fatty acids; SFA, saturated fatty acid; MUFA, monounsaturated fatty acid; PUFA, polyunsaturated fatty acid; HMLE, hard-shelled mussel lipid extract; ND, not detected or negligible.

### 3.2. Patient Characteristics

**Figure 1:**
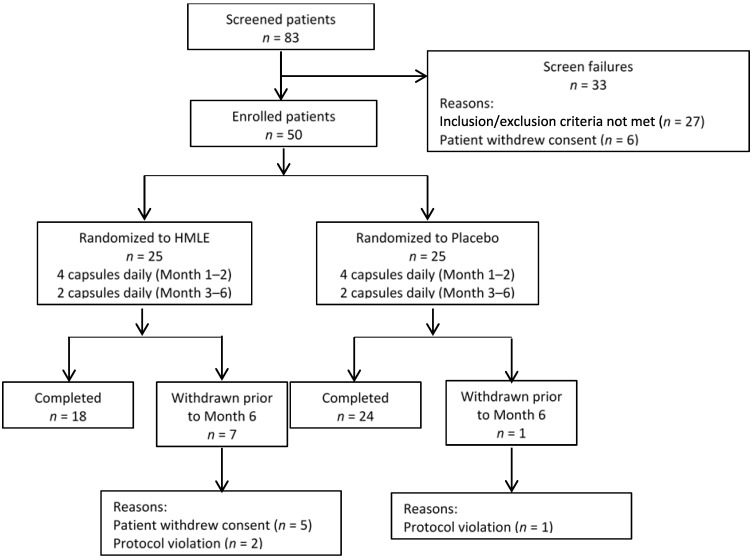
Flowchart of patient disposition over 6 months.

The first patient was screened in December 2013 and the final evaluation was performed in August 2014. A total of 50 patients with active RA entered into this study and were randomized 1:1 to receive either HMLE or placebo (Figure 1). Among the enrolled patients, eight patients did not complete the study: one male and four female patients in HMLE group who complained the flavors of capsules after burp and high transportation costs, rejected to visit our outpatient clinic at month 3 or month 6; two female patients in HMLE group and one male patient in placebo were considered withdrawn from the study as they reported that they suspended taking capsules for 4–6 weeks. Finally, 42 participants (12 males, 30 females) completed the 6-month study and the mean age was 58 (range = 28–75 year). In the ITT analysis, patients lacking month 3 data and month 6 data were considered as treatment failures.

As shown in Table 2, most prescribed medicines being used during the study in both groups were methotrexate, corticosteroid (mainly methylprednisolone and prednisone) and Chinese patent medicine (mainly thunder god vine and total glucosides of paeony), and the distribution of these medicines were comparable between groups. Additional details of completers’ baseline characteristics were summarized in Table 2, which indicated no statistically significant difference between the two groups at entry. By comparing the baseline characteristics between completers and those who dropped, the drop-outs (two males and six females) had higher patient global scores (56.9 *vs.* 50.3, *p* < 0.05); however, they had lower swollen joint counts (4.5 *vs.* 7.5, *p* < 0.05). No significant difference was found in other baseline characteristics between completers and drop-outs. There was no serious adverse event related to oil supplementation observed in both groups throughout the study. For the parameters in relation to liver and renal function, elevated alanine aminotransferase (1.5 times the upper limits of normal < ALT< 3 times the upper limits of normal) was observed for two patients in HMLE group and three patients in placebo group. Additionally, aspartate aminotransferase elevation (1.5 times the upper limits of normal < AST< 3 times the upper limits of normal) was observed for one patient in HMLE group and two patients in placebo group. Data of liver and renal function tests before and after treatment were presented in Table 3. Even though a few participants reported that they have experienced slight nausea, vomiting and loss of appetite during the study (Table 4), no significant difference in frequency was observed between groups and it was not certain if these symptoms were related to oil supplementation. Table 5 summarized the dietary intakes of completers before intervention and during week 12. Except that total fat intake was significantly increased in both groups which may be due to the oil supplementation, no other significant changes in daily dietary nutrients and energy intakes were found.

### 3.3. Improvement in Disease Activity

**Figure 2:**
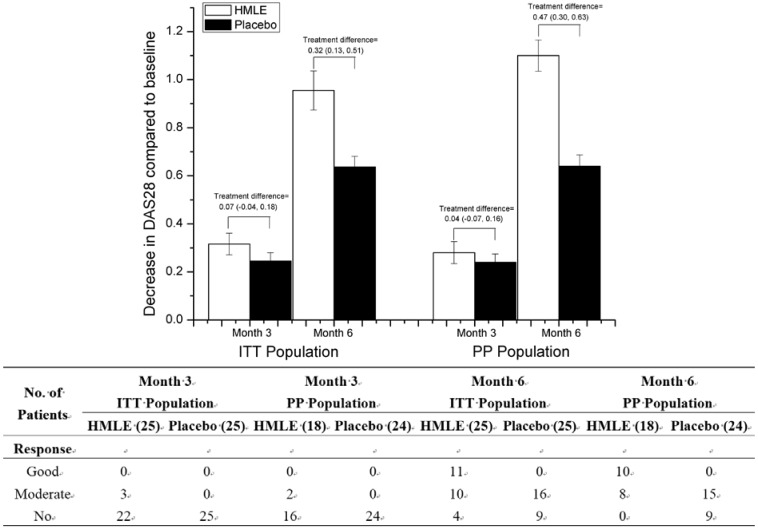
Changes over time in the Disease Activity Score (DAS28), and number of patients achieving good or moderate response to intervention based on DAS28. Error bars represent SEM.

The mean improvements on DAS28 in both HMLE group and placebo group are shown in Figure 2. In the ITT population, treatment difference is 0.07 (95% CI: −0.04, 0.18) after 3 months and 0.32 (95% CI: 0.13, 0.51) after 6 months, which confirmed the unequivalence between HMLE and placebo groups. While in PP population, treatment difference is 0.04 (95% CI: −0.07, 0.16) and 0.47 (95% CI: 0.30, 0.63) after 3 months and 6 months, respectively. Even though no more than three patients in the HMLE group showed moderate DAS28 response after 3 months’ intervention, most patients showed good or moderate DAS28 response at the end of the study (84% in ITT population and 100% in PP population). For patients in placebo group who obtained moderate DAS28 response at the end, the amount was almost the same between ITT population (16 of 25 patients) and PP population (15 of 24 patients), with others obtaining no response.

**Figure 3:**
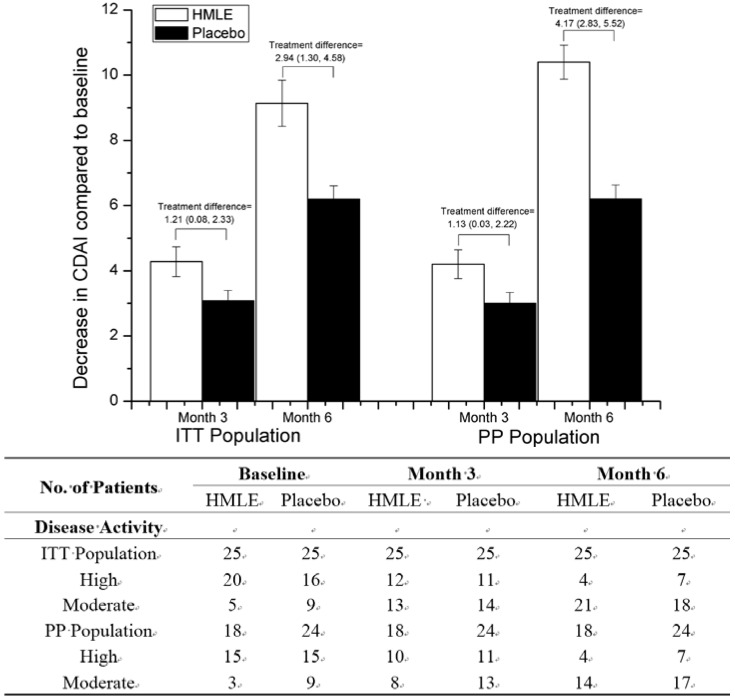
Changes over time in the Clinical Disease Activity Index (CDAI), and number of patients classified in High or Moderate severity of disease activity based on CDAI. Error bars represent SEM.

With regard to clinical disease activity index (CDAI), similar trends were noted and the changes in CDAI over time of both groups were shown in Figure 3. Both groups exhibited a significant reduction in CDAI over time (*p* < 0.01 for both time points), however, patients in HMLE group showed much larger reduction at the end of the study compared with that in placebo group (treatment difference 2.94, 95% CI: 1.30, 4.58 in ITT population; treatment difference 4.17, 95% CI: 2.83, 5.52 in PP population). According to the CDAI, 20 ITT patients and 15 PP patients in HMLE group were classified as severe grade with CDAI > 22, 16 of the 20 severe-grade ITT patients (80%) and 11 of the 15 severe-grade PP patients (73%) recovered to moderate grade after 6 months, respectively. In placebo group, however, only 9 of 16 severe-grade ITT patients (56%) and 8 of 15 severe-grade PP patients (53%) recovered to moderate grade after treatment, respectively. 

For other clinical indicators of disease activity, such as tender joint count based on 28-joint assessment (TJC), swollen joint count based on 28-joint assessment (SJC), morning stiffness, physician global assessment and patient global assessment (100 mm visual analog scale), the changes after 3 months or 6 months from baseline were all presented in Table 6. Repeated measure ANOVA analysis showed time effect was significant for these clinical indicators, which indicated there were significant improvements in both groups after intervention. Significant time and group interaction for TJC, SJC and morning stiffness indicated significant larger degree of improvements that favored HMLE group. However, no significant difference in the declining trend was found between groups with respect to physician global assessment and patient global assessment of disease activity. With regard to laboratory indicators including ESR, CRP and RF, significant decrease was observed in both HMLE and placebo groups, while no significant between-groups difference was found.

**Table 2:** Baseline characteristics of subjects completing the study who represents the per-protocol population *.

Variables	HMLE Group			Placebo Group			*p*-Value ^a^
	Total (*n* = 18)	Men (*n* = 6)	Women (*n* = 12)	Total (*n* = 24)	Men (*n* = 6)	Women (*n* = 18)	
*Subject characteristics*							
Age (years)	56.6 ± 2.8	60.5 ± 2.6	54.7 ± 4.0	58.3 ± 2.18	57.7 ± 4.4	58.4 ± 2.6	0.642
BMI (kg/m^2^)	22.4 ± 0.60	23.3 ± 1.2	22.0 ± 0.65	22.5 ± 0.42	23.0 ± 0.83	22.4 ± 0.50	0.863
SBP (mmHg)	118.9 ± 3.4	129.7 ± 3.7	113.6 ± 4.0	124.0 ± 2.7	133.2 ± 3.0	120.9 ± 3.1	0.246
DBP (mmHg)	76.6 ± 2.3	84.0 ± 3.7	72.9 ± 2.3	80.3 ± 1.5	87.3 ± 1.8	78.0 ± 1.5	0.163
Duration of RA (years)	7.1 ± 0.76	7.8 ± 0.63	6.7 ± 1.10	8.0 ± 0.87	5.4 ± 0.90	8.8 ± 1.1	0.447
Total number of tender joints	10.6 ± 0.44	10.5 ± 0.56	10.7 ± 0.60	9.5 ± 0.60	8.5 ± 0.67	9.8 ± 0.76	0.150
Total number of swollen joints	7.3 ± 0.69	8.3 ± 1.4	6.8 ± 0.74	7.8 ± 0.73	7.7 ± 1.6	7.8 ± 0.85	0.623
Morning stiffness (minutes)	69.7 ± 6.3	67.5 ± 13.3	70.8 ± 7.1	72.7 ± 8.0	66.7 ± 10.2	74.7 ± 10.2	0.782
ESR (mm/h)	50.8 ± 6.4	72.3 ± 9.5	40.0 ± 6.6	53.3 ± 6.1	47.8 ± 14.3	55.1 ± 6.9	0.785
CRP (mg/dL)	14.4 ± 1.7	16.0 ± 4.4	13.7 ± 1.5	16.3 ± 2.0	11.9 ± 3.5	17.8 ± 2.3	0.490
DAS(28)	5.80 ± 0.12	6.18 ± 0.09	5.60 ± 0.15	5.71 ± 0.15	5.47 ± 0.35	5.79 ± 0.16	0.657
Physician global (0–100 mm)	42.9 ± 2.6	44.0 ± 4.3	42.4 ± 3.3	40.9 ± 2.8	41. 7 ± 4.8	40.7 ± 3.4	0.605
Patient global (0–100 mm)	53.5 ± 2.7	55.0 ± 4.3	52.8 ± 3.6	47.9 ± 2.6	48.7 ± 4.8	47.6 ± 3.2	0.153
CDAI	27.5 ± 1.4	28.7 ± 2.4	26.9 ± 1.8	26.1 ± 1.6	25.2 ± 2.9	26.4 ± 2.0	0.534
*Patients taking medicines*							
Methotrexate	15	5	10	19	8	11	0.734
Corticosteroid	9	4	5	13	5	8	0.789
NSAIDS	4	2	2	6	2	4	0.834
Chinese patent medicine	9	5	4	11	5	6	0.789
TNF blockers	2	0	2	2	1	1	0.762

* No significant difference was found in the baseline characteristics between original intention-to-treat population and per-protocol population; ^a^ *p*-value for between-groups difference; Data are presented as mean ± SEM.

**Table 3:** Values of liver and renal function variables before and after treatment.

Variables	HMLE (18)			Placebo (24)			*p* Value		
	Month 0	Month 6	Difference (95% CIs)	Month 0	Month 6	Difference (95% Cis)	Time	Group	Time × Group
TBIL (µmol/L)	14.09 ± 1.38	11.98 ± 1.39	−2.11 (−4.84, 0.62)	13.50 ± 0.91	13.56 ± 1.17	0.054 (−3.40, 3.51)	0.363	0.704	0.338
DBIL (µmol /L)	4.11 ± 0.42	3.77 ± 0.53	−0.34 (−1.44, 0.76)	3.83 ± 0.33	4.97 ± 0.50	1.14 (−0.018, 2.30)	0.316	0.370	0.068
IBIL (µmol /L)	9.98 ± 1.15	8.21 ± 0.95	−1.77 (−3.54, 0.03)	9.68 ± 0.66	8.59 ± 0.73	−1.09 (−3.45, 1.27)	0.065	0.972	0.652
ALT (U/L)	26.44 ± 2.63	26.11 ± 5.20	−0.33 (−9.32, 8.66)	23.50 ± 2.37	27.13 ± 4.59	3.63 (−2.04, 9.29)	0.502	0.847	0.420
AST (U/L)	27.22 ± 1.84	27.56 ± 3.41	0.33 (−8.20, 8.86)	23.67 ± 3.56	25.00 ± 3.22	1.33 (−9.32, 11.99)	0.197	0.890	0.246
γ-GT (U/L)	20.00 ± 2.37	16.78 ± 1.40	−3.22 (−7.36, 0.92)	24.58 ± 2.66	25.50 ± 1.93	0.92 (−5.24, 7.07)	0.551	0.012	0.287
TP (g/L)	78.67 ± 1.18	78.79 ± 0.89	0.033 (−3.30, 3.37)	78.65 ± 0.76	78.78 ± 0.82	0.12 (−1.79, 2.03	0.930	0.974	0.960
ALB (g/L)	48.04 ± 0.67	48.44 ± 0.60	0.39 (−1.64, 2.43)	47.58 ± 0.39	45.66 ± 0.53	−0.92 (−2.16, 0.32)	0.629	0.048	0.231
GLB (g/L)	30.26 ± 0.68	30.62 ± 0.92	−0.36 (−2.67, 1.94)	31.07 ± 0.69	32.11 ± 0.57	1.04 (−0.66, 2.74)	0.641	0.138	0.302
BUN (mmol/L)	5.58 ± 0.25	5.30 ± 0.26	−0.28 (−1.06, 0.49)	5.23 ± 0.31	5.34 ± 0.37	0.11 (−0.63, 0.85)	0.745	0.667	0.457
Cr (mmol/L)	64.21 ± 3.30	68.55 ± 3.33	4.34 (−5.49, 14.17)	66.61 ± 2.68	63.08 ± 3.39	−3.53 (−10.72, 3.65)	0.887	0.668	0.174
UA (mmol/L)	319.8 ± 22.3	280.5 ± 17.3	−39.3 (−97.9, 19.4)	296.4 ± 16.1	294.1 ± 18.9	−2.2 (−49.3, 44.9)	0.305	0.807	0.251

TBIL, total bilirubin; DBIL, direct bilirubin; IBIL, indirect bilirubin; ALT, alanine aminotransterase; AST, aspartate aminotransferase; γ-GT, gamma-glutamyl transpeptidase; TP, total protein; ALB, albumin; GLB, globulin; BUN, blood urea nitrogen; Cr, creatinine; UA, uric acid.

**Table 4:** Adverse events reported during the study period.

Adverse Events	HMLE Group (18)	Placebo Group (24)	*p*-Value ^a^
Elevated ALT	2	3	0.891
Elevated AST	1	2	0.729
Loss of appetite	8	12	0.721
Nausea	5	7	0.921
Vomiting	4	5	0.914

^a^ *p*-value for between-group differences. ALT, alanine aminotransterase; AST, aspartate aminotransferase.

**Table 5:** Dietary intakes at baseline and at week 12 of participants completing the study.

Variables	HMLE Group (*n* = 18)		Placebo Group (*n* = 24)		*p* Value		
	Week 0	Week 12	Week 0	Week 12	Time	Group	Time × Group
Total energy (kcal)	1858.9 ± 40.6	1843.8 ± 41.6	1846.5 ± 44.5	1824.2 ± 38.6	0.072	0.788	0.726
Carbohydrate (g)	261.0 ± 8.4	259.4 ± 7.6	269.5 ± 9.0	265.3 ± 8.6	0.199	0.557	0.558
Total fat (g)	52.6 ± 1.0	54.1 ± 1.4 *	49.9 ± 1.2	51.3 ± 1.2 *	0.003	0.117	0.832
Protein (g)	85.0 ± 4.1	79.7 ± 6.0	79.1 ± 4.6	75.3 ± 4.1	0.061	0.42	0.759
Carbohydrate (% of energy)	55.9 ± 0.88	56.1 ± 0.10	58.1 ± 0.78	57.9 ± 0.97	0.956	0.126	0.584
Fat (% of energy)	25.8 ± 0.78	26.7 ± 0.92 *	24.9 ± 0.80	25.6 ± 0.75 *	0.001	0.358	0.705
Protein (% of energy)	18.3 ± 0.66	17.1 ± 1.0	17.1 ± 0.87	16.6 ± 0.85	0.073	0.445	0.512

Data are presented as mean ± SEM; * *p* < 0.05, compared to week 0 for each intervention.

### 3.4. Effect of HMLE Supplementation on Blood Levels of Fatty Acids Composition and Inflammatory Mediators and Cytokines

**Figure 4:**
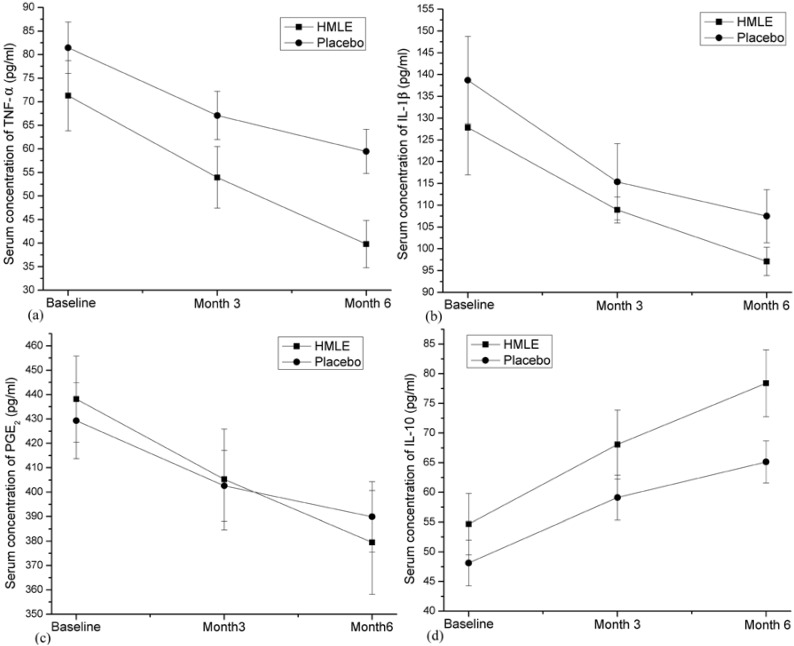
Changes over time in serum concentration of TNF-α (**a**); IL-1β (**b**); PGE_2_ (**c**) and IL-10 (**d**). Error bars represent SEM.

To investigate whether the HMLE supplementation affected the blood levels of fatty acids composition and balance between pro-inflammatory and anti-inflammatory mediators which may regulates pathogenesis of RA. Fatty acids composition in erythrocyte membrane and TNF-α, IL-1β, PGE2, IL-6 and IL-10 in serum were measured for patients in each group. The fatty acids’ compositions before and after treatments were presented in Table 7 and the inflammatory mediators’ concentrations at each time point of measurement were summarized in Table 6. For *n*-3 PUFAs (C22:6*n*-3 and C20:5*n*-3) which were determined in larger amounts in HMLE, a significant increase (*p* < 0.05) in erythrocyte membrane was observed in patients receiving HMLE capsules, while no significant changes were found among patients receiving placebo capsules. Even though linoleic acid (C18:2*n*-6), oleic acid (C18:1*n*-9) and palmitic acid (C16:0) account for almost 95% of all fatty acid compositions in placebo capsules, the increase of these fatty acid compositions in erythrocyte membrane was only significant for linoleic acid (C18:2*n*-6) in placebo group after intervention (*p* < 0.01). Serum TNF-α at baseline ranged from 32.2 to 131.1 (71.3 ± 7.4 pg/mL) in HMLE group and 33.2–138.9 (81.5 ± 5.5 pg/mL) in placebo group. Serum IL-1β at baseline was between 61 and 225 (127.8 ± 10.8 pg/mL) in HMLE group and between 68 and 227 (138.7 ± 10.0 pg/mL) in placebo group. As shown in Figure 4a,b, both serum TNF-α and IL-1β decreased significantly in both groups after 3 months and 6 months from the baseline. Moreover, the decrease of TNF-α in HMLE group after 6 months was more evident than that in placebo group (−31.5 ± 4.2 pg/mL *vs.* −22 ± 1.8 pg/mL, *p* < 0.05), while no significant differences were found in degree of IL-1β decrease between both groups (−30.7 ± 8.9 pg/mL *vs.* −31.2 ± 5.9 pg/mL, *p* = 0.963). For the changes of serum PGE_2_ level, similar results to that of TNF-α were found, which indicate significant reduction within the group after 3 months or 6 months from baseline and a significantly larger decrease in the HMLE group after 6 months (−58.7 ± 5.2 *vs.* −39.4 ± 2.9, *p* < 0.01; Figure 4c). However, serum IL-6 levels in the present study did not show any significant changes after 3 months or 6 months from baseline in both groups, and no between-group difference was observed (Table 6). For IL-10, which was a key anti-inflammatory cytokine involved in arthritic diseases, a significant increase was observed after 3 months and 6 months in both groups. Notably, after 6 months from baseline, the degree of increase in the HMLE group was significantly larger than that in placebo group (23.7 ± 2.2 pg/mL *vs.* 17.0 ± 1.6 pg/mL, *p* < 0.05; Figure 4d).

**Table 6:** Values of assessed clinical indicators for disease activity before and after intervention.

Variables	HMLE Group (*n* = 18)			Placebo Group (*n* = 24)			*p* Value		
	Month 0	Month 3	Month 6	Month 0	Month 3	Month 6	Time	Group	Time × Group
SBP (mmHg)	118.9 ± 3.4	118.2 ± 2.2	121.4 ± 2.8 *	124.0 ± 2.7	124.4 ± 2.0	122.8 ± 2.4	0.610	0.238	0.056
DBP (mmHg)	76.6 ± 2.3	75.7 ± 1.5	78.4 ± 1.9	80.3 ± 1.5	81.0 ± 1.4	78.7 ± 1.1	0.944	0.143	0.007
TJC (28)	10.6 ± 0.4	8.7 ± 0.5 **	5.1 ± 0.5 **	9.5 ± 0.6	8.2 ± 0.7 **	6.9 ± 0.5 **	<0.01	0.917	<0.01
SJC (28)	7.3 ± 0.7	5.9 ± 0.5 **	4.1 ± 0.5 **	7.8 ± 0.73	6.5 ± 0.7 **	5.3 ± 0.6 **	<0.01	0.417	0.053
Morning stiffness (min)	69.7 ± 6.3	51.1 ± 7.7 **	40.6 ± 7.0 **	72.7 ± 8.0	62.9 ± 7.6 **	58.1 ± 6.6 **	<0.01	0.296	0.016
ESR	50.8 ± 6.4	48.0 ± 5.3	34.7 ± 4.7 **	53.3 ± 6.1	49.2 ± 4.8	38.9 ± 3.9 **	<0.01	0.719	0.571
CRP	14.4 ± 1.7	12.0 ± 1.0 *	11.4 ± 1.5 **	16.3 ± 2.0	15.1 ± 1.8	14.9 ± 1.6 *	<0.01	0.236	0.273
RF	204.6 ± 33.1	196.1 ± 24.6	183.7 ± 23.8	211.7 ± 23.0	204.1 ± 22.8	201.6 ± 20.9	0.057	0.743	0.650
DAS(28)	5.80 ± 0.12	5.51 ± 0.11 **	4.69 ± 0.12 **	5.71 ± 0.15	5.46 ± 0.14 **	5.07 ± 0.14 **	<0.01	0.678	<0.01
Physician global	42.9 ± 2.6	40.4 ± 2.3 *	37.2 ± 2.2 **	40.9 ± 2.8	39.4 ± 2.5	36.4 ± 2.1 **	<0.01	0.707	0.753
Patient global	53.5 ± 2.7	47.7 ± 2.2 **	42.4 ± 2.3 **	47.9 ± 2.6	44.8 ± 2.2 *	40.6 ± 2.1 **	<0.01	0.288	0.135
CDAI	27.5 ± 1.4	23.4 ± 1.2 **	17.2 ± 1.3 **	26.1 ± 1.6	23.1 ± 1.6 **	19.9 ± 1.4 **	<0.01	0.861	<0.01
*Inflammatory factors*									
TNF-α	71.3 ± 7.4	53.9 ± 6.6 **	39.8 ± 5.0 **	81.5 ± 5.5	67.1 ± 5.1 **	59.5 ± 4.7 **	<0.01	0.076	0.029
IL-1β	127.8 ± 10.8	108.9 ± 3.0 *	97.1 ± 3.3 **	138.7 ± 10.0	115.4 ± 8.8 **	107.5 ± 6.1 **	<0.01	0.377	0.739
IL-6	109.4 ± 5.8	109.9 ± 4.6	106.4 ± 4.1	96.7 ± 8.8	95.4 ± 9.0	94.4 ± 9.1	0.130	0.245	0.617
IL-10	54.7 ± 5.2	68.1 ± 5.8 **	78.4 ± 5.6 **	48.1 ± 3.8	59.1 ± 3.8 **	65.1 ± 3.6 **	<0.01	0.138	0.014
PGE2	438.2 ± 17.7	405.2 ± 20.6 **	379.5 ± 21.2 **	429.3 ± 15.5	402.6 ± 14.5 **	389.9 ± 14.4 **	<0.01	0.988	<0.01

* *p* < 0.05, compared to baseline for each intervention; ** *p* < 0.01, compared to baseline for each intervention. SBP, systolic pressure; DBP, diastolic pressure; TJC, tender joint count; SJC, swollen joint count; ESR, erythrocyte sedimentation rate; CRP, C-reactive protein; RF, rheumatoid factor; DAS28, disease activity score; CDAI, clinical disease activity index; TNF-α, tumor necrosis factor α; IL-1β, interleukin-1β; IL-6, interleukin-6; IL-10, interleukin-10; PGE2, prostaglandin E2.

**Table 7:** Fatty acid composition in erythrocyte membranes (% total fatty acids) of participants before and after intervention.

	HMLE Group (*n* = 18)			Placebo Group (*n* = 24)			*p* Value		
	M0	M6	Difference (95% CIs)	M0	M6	Difference (95% CIs)	Time	Group	Time × Group
C14:0	0.12	0.14	0.017 (−0.010,0.045)	0.10	0.13	0.026 (−0.039, 0.091)	0.101	0.155	0.745
C15:0	0.06	0.08	0.019 (0.007, 0.033) **	0.06	0.06	0.001 (−0.014, 0.016)	0.067	0.053	0.095
C16:0	21.40	22.97	1.57 (−0.30, 3.44)	21.03	23.15	2.12 (−0.73, 4.96)	0.032	0.909	0.734
C17:0	0.22	0.28	0.064 (0.031, 0.097) **	0.19	0.22	0.029 (−0.024, 0.082)	<0.01	0.018	0.228
C18:0	15.35	14.93	−0.42 (−1.79,0.95)	14.72	14.78	0.062 (−4.77, 4.89)	0.808	0.514	0.745
C20:0	0.29	0.37	0.081 (−0.039, 0.20)	0.25	0.45	0.21 (−0.060, 0.47)	0.018	0.700	0.276
C22:0	0.62	0.63	0.012 (−0.11, 0.13)	0.63	0.66	0.035 (−0.21, 0.28)	0.663	0.851	0.833
C24:0	1.69	1.70	0.014 (−0.47, 0.50)	1.80	1.29	−0.51 (−1.05, 0.076)	0.186	0.507	0.165
SFA	39.76	41.12	1.36 (−2.06, 4.77)	38.79	40.72	1.93 (−3.47, 7.30)	0.277	0.652	0.851
C16:1*n*-7	0.12	0.14	0.019 (−0.018, 0.056)	0.12	0.15	0.027 (−0.027, 0.081)	0.162	0.998	0.791
C18:1*n*-9	14.23	13.62	−0.60 (−1.81, 0.61)	13.90	15.67	1.77 (−1.25, 4.79)	0.637	0.502	0.344
C20:1*n*-9	0.49	0.36	−0.14 (−0.22, −0.058) **	0.48	0.45	−0.028 (−0.36, 0.30)	0.089	0.458	0.249
C22:1*n*-9	0.38	0.20	−0.170 (−0.36, −0.01) *	0.28	0.30	0.016 (−0.45, 0.48)	0.349	0.982	0.267
C24:1*n*-9	4.63	4.18	−0.45 (−1.31, 0.42)	4.53	3.81	−0.72 (−2.47, 1.03)	0.147	0.625	0.728
MUFA	19.86	18.51	−1.35 (−3.06, 0.37)	19.31	20.38	1.07 (−3.39, 5.52)	0.912	0.669	0.352
C18:3*n*-3	0.15	0.16	0.0027 (−0.028, 0.033)	0.16	0.13	−0.032 (−0.11, 0.044)	0.321	0.784	0.242
C20:3*n*-3	0.11	0.08	−0.038 (−0.071, −0.005) *	0.10	0.06	−0.038 (−0.14, 0.062)	0.035	0.414	0.993
C20:5*n*-3	0.59	1.36	0.77 (0.39, 1.16) **	0.45	0.59	0.14 (−0.18, 0.46)	<0.01	0.017	0.057
C22:5*n*-3	2.01	2.25	0.24 (−0.18, 0.67)	2.22	1.36	−0.86 (−1.57, −0.16) *	0.107	<0.01	0.214
C22:6*n*-3	4.29	5.23	0.94 (0.041, 2.27) *	4.38	3.58	−0.80 (−2.9, 1.29)	0.891	0.160	0.017
*n*-3 PUFA	7.14	9.07	1.92 (0.091, 3.76) *	7.31	5.73	−1.59 (−4.26, 1.08)	0.831	0.101	0.036
C18:2*n*-6	12.65	13.79	1.13 (−0.65, 2.92)	12.34	16.45	4.11 (0.77, 7.59) **	<0.01	0.196	0.102
C18:3*n*-6	0.03	0.02	−0.0063 (−0.029, 0.017)	0.02	0.03	0.011 (−0.023, 0.045)	0.804	0.858	0.380
C20:2*n*-6	0.34	0.29	−0.041 (−0.097, 0.015)	0.31	0.32	0.0077 (−0.18, 0.19)	0.573	0.919	0.411
C20:3*n*-6	1.42	1.30	−0.12 (−0.43, 0.20)	1.73	1.55	−0.18 (−1.12, 0.75)	0.352	0.055	0.835
C20:4*n*-6	16.07	14.02	−2.05 (−4.19, 0.095)	17.02	13.12	−3.90 (−6.79, 1.99)	0.015	0.982	0.410
C22:2*n*-6	0.10	0.09	−0.0088 (−0.075, 0.057)	0.04	0.09	0.051 (−0.11, 0.21)	0.495	0.338	0.569
C22:4*n*-6	2.64	1.79	−0.85 (−1.17, −0.53) **	3.13	1.64	−1.49 (−2.46, −0.51) *	<0.01	0.489	0.065
*n*-6 PUFA	33.23	31.30	−1.93 (−5.62, 1.76)	34.59	33.20	−1.39 (−4.64, 1.86)	0.289	0.350	0.860

* *p* < 0.05, compared to baseline for each intervention; ** *p* < 0.01, compared to baseline for each intervention.

## 4. Discussion

Even though previous studies [21,22,23] focusing on New Zealand green-lipped mussel lipid extract (GLME) have revealed the benefits for patients with arthritis, the present study is the first randomized, double-blind, placebo-controlled trial evaluating the efficacy of Chinese hard-shell mussel lipid extract in improving disease activity in RA patients. Although the appearance of HMLE capsules and placebo capsules used in the present study was the same, they could be distinguished by the taste after a reflux. However, none of the patients had previously experienced HMLE and none of them had a chance to compare HMLE capsules and placebo capsules; it is unlikely that they would know which preparation they were given. The dose of HMLE used in the present study (800 mg/day for the first 2 months and 400 mg/day thereafter) was comparable with that of GMLE generally used in previous studies. The objective of higher dose (four capsules) during the first 2 months was intended to induce a sharp increase of potential active compound concentration in tissues. After the first 2 months, the dosage was reduced to two capsules aimed at maintaining the potential active compound concentration in tissues. A similar schedule was also reported elsewhere [23,24]. It is noteworthy that the dose of HMLE here was much lower than that of fish oil supplementation recommended for patients with RA [25].

The overall safety profiles of both HMLE and placebo groups were similar. As high frequency of nausea and anorexia was observed in both groups comparably, the background medicines including methotrexate, methylprednisolone and prednisone, which were widely used in both groups, may help explain the adverse events. Moreover, the observed ALT and AST elevation may also be related to the background drugs which have been reported to be related to adverse events such as elevated plasma liver enzyme levels [26,27]. Overall, the HMLE in the present study was safe, as no significant between-group difference regarding the adverse events was observed. These results were consistent with the previously reported well-tolerance and non-gastrotoxic properties of HMLE in animal models and GMLE in humans [14,22,23].

The primary endpoint was met by demonstrating that DAS28 decreased significantly in both groups after 3 months and 6 months from baseline, which also implied the efficacy of background prescribed medicines. Nevertheless, the decrease in DAS28 at month 6 for patients receiving HMLE was significantly larger than that of patients receiving placebo, and significant treatment differences were observed in both the ITT population and PP population which may help to strengthen the certainty of results despite the high dropout rate. Similar results were also observed for CDAI, morning stiffness, total tender joint counts and total swollen joint counts, indicating the responses to HMLE were more significant at months 6 than months 3. Therefore, the present results may indicate that HMLE have a long-term effect on improvement of RA. This was also consistent to previous studies evaluating anti-arthritic effects of Green-lipped mussel [22,28], which lacked significant difference in mean outcome measures before 3 months of treatment. Therefore, marine nutritional supplements, such as GMLE and HMLE, may have synergistic effects with prescribed drugs on RA and tend to exhibit their benefits in the long run. Any future trials of fish oil, HMLE and GMLE shorter than 3 months may have limited value. The present study also observed significant increase of blood n-3 fatty acids in patients receiving HMLE capsules after 6 months, which accorded with the high content of docosahexenoic acid and eicosapentaenoic acid in HMLE. However, the anti-inflammatory effects could not be solely attributed to *n*-3 fatty acids, because previous study [29] indicated that New Zealand green-lipped mussel lipid extract (GMLE), containing much lower content of n-3 PUFA than that of tuna oil, exhibited much higher anti-inflammatory activity than tuna oil. Moreover, our study has proven similarly strong anti-inflammatory activity of HMLE compared with GMLE in rat models [14]. Therefore, the synergistic effects may also exist between n-3 PUFA and other potential active compounds or among different lipid classes of HMLE, which could be partially proven in our previous study evaluating the anti-inflammatory activity of single lipid class of HMLE separately in rats with granuloma swelling. We found that the summative inhibition rate of each single lipid class was much lower than that of whole HMLE (unpublished data). A further study on identifying active compounds in HMLE is underway and will be reported in due course.

Regarding mechanisms behind the efficacy in relieving RA symptoms, modulation in the balance between pro- and anti-inflammatory mediators played an important role. TNF is a potent pro-inflammatory factor and of primary importance in pathogenesis of rheumatoid arthritis [30,31], and the inhibition of TNF could suppress various arthritis models while overexpression of TNF could induce spontaneous erosive inflammatory arthritis [32]. IL-6, as well as TNF, IL-1β and PGE2, were reported to up-regulate the RANKL (receptor activator of NF-κB ligand) expression [33,34], which was an important prerequisite for osteoclast differentiation and arthritis [35,36,37]. Therapeutic blockade of TNF could yield rapid decrease of plasma IL-6 level [38], and our previous study based on rat model of arthritis has revealed that HMLE strongly inhibited the excessive production of both TNF and IL-6 [14]. However, in the present study, only TNF but not IL-6 in serum was observed to be significantly decreased. One explanation is that TNF does not drive downstream cytokines such as IL-6 and IL-1 in a linear model which was proposed originally, and parallel cytokine pathways with ongoing crosstalk at multiple levels are more likely [11,30]. Additionally, previous studies have also observed synergistic effects between IL-1β and TNF, which together lead to synovial fibroblast activation and cytokine production [39]. Notably, both TNF and IL-1β were reported to be correlated with the synovial expression of IL-32, which could promote PGE2 synthesis *in vitro* [40], and freeze-dried green-lipped mussel powder was found to exhibit prostaglandin biosynthesis inhibitory activity in rats [41]. In the present study, serum IL-1β was observed to be significantly decreased within each group and no significant difference was observed between groups. For serum PGE2 concentration, much more evident decrease was observed in the HMLE group compared with that in the placebo group. Therefore, the pathway that TNF and IL-1β promote PGE2 synthesis may help explain the accompanying decrease of serum PGE2 with TNF and IL-1β. Moreover, the n-3 fatty acid contained in HMLE, which had been reported to decrease prostaglandin synthesis by acting as inhibitors of membrane arachidonic acid metabolism via the cyclooxygenase (COX) and lipoxygenase (LOX) pathways [29,42,43], may help explain the much more evident decrease of PGE2 in the HMLE group. There are some limitations in the present study. Firstly, this study was a single-center trial with small sample size. Secondly, dropout rate for HMLE group was relatively high. Nevertheless, ITT analysis was performed to provide a conservative explanation. Thirdly, patients’ prescribed medicines were permitted because it was unethical to treat RA patients with only HMLE or placebo for 6 months. Since no significant difference in distributions of background medicines (mainly Methotrexate, Corticosteroids, Chinese patent medicines and NSAIDs) was observed between groups, and therefore, more evident improvement in the HMLE group may still be qualified to prove efficacy. Fourthly, all patients were allowed to decrease the dose of traditional treatment (prednisolone and NSAIDs) flexibly when they felt good or unacceptable side-effects were showing, but exact increased/decreased dose of these medications was not evaluated. Lastly, subjects were asked to maintain their regular physical activity, dietary habits and overall lifestyle during the period, but incompliance could have possibly occurred.

## 5. Conclusions

In conclusion, present results suggest that HMLE supplementation was safe and had significant beneficial effects in improving clinical disease activity of patients with active rheumatoid arthritis. The improvement in the balance between pro- and anti-inflammatory factors may serve as the mechanisms underlying efficacy. These results highlighted that HMLE could be a good supplement for RA patients on the basis of original therapy. However, more multi-center and long-term studies with larger sample sizes are needed to confirm our observations.
